# An Intelligent Sensor Based Decision Support System for Diagnosing Pulmonary Ailment through Standardized Chest X-ray Scans

**DOI:** 10.3390/s22197474

**Published:** 2022-10-02

**Authors:** Shivani Batra, Harsh Sharma, Wadii Boulila, Vaishali Arya, Prakash Srivastava, Mohammad Zubair Khan, Moez Krichen

**Affiliations:** 1 Department of Computer Science and Engineering, KIET Group of Institutions, Ghaziabad 201206, India; 2 Robotics and Internet-of-Things Laboratory, Prince Sultan University, Riyadh 12435, Saudi Arabia; 3 RIADI Laboratory, National School of Computer Sciences, University of Manouba, Manouba 2010, Tunisia; 4 School of Engineering, GD Goenka University, Gurugram 122103, India; 5 Department of Computer Science and Engineering, Graphic Era (Deemed to Be University), Dehradun 248002, India; 6 Department of Computer Science and Information, Taibah University, Medina 42353, Saudi Arabia; 7 Faculty of Computer Science & IT, Al Baha University, Al Baha 65779, Saudi Arabia

**Keywords:** chest X-ray scans, COVID-19, decision support system, deep leaning, pneumothorax

## Abstract

Academics and the health community are paying much attention to developing smart remote patient monitoring, sensors, and healthcare technology. For the analysis of medical scans, various studies integrate sophisticated deep learning strategies. A smart monitoring system is needed as a proactive diagnostic solution that may be employed in an epidemiological scenario such as COVID-19. Consequently, this work offers an intelligent medicare system that is an IoT-empowered, deep learning-based decision support system (DSS) for the automated detection and categorization of infectious diseases (COVID-19 and pneumothorax). The proposed DSS system was evaluated using three independent standard-based chest X-ray scans. The suggested DSS predictor has been used to identify and classify areas on whole X-ray scans with abnormalities thought to be attributable to COVID-19, reaching an identification and classification accuracy rate of 89.58% for normal images and 89.13% for COVID-19 and pneumothorax. With the suggested DSS system, a judgment depending on individual chest X-ray scans may be made in approximately 0.01 s. As a result, the DSS system described in this study can forecast at a pace of 95 frames per second (FPS) for both models, which is near to real-time.

## 1. Introduction

COVID-19 has unexpectedly emerged as a worldwide epidemic [1]. Individuals with a previously undiscovered lung ailment were discovered around the end of the year 2019 [2]. After the first sufferer was hospitalised, the identification of COVID-19 was verified in more than 1975 additional people within a month. COVID-19 is triggered by a novel coronavirus known as SARS-CoV-2 (severe acute respiratory syndrome coronavirus) [2,3]. The COVID-19 epidemic has intensified significant pressure on most nations’ healthcare systems, essential services, and finances [4,5,6]. COVID-19 has killed people throughout the world [7,8]. Real-time reverse-transcription polymerase chain reaction (RT-PCR) is the most frequently utilized diagnostic technique for COVID-19 to date [9].

The primary screening methods for detecting and diagnosing pulmonary chest disorders early in the diagnostic workup, along with COVID-19, are radiographic imaging methods such as chest digitized X-ray (CXR) and computed tomography (CT) [1,10,11]. CT has demonstrated superiority over CXR [12,13]. Radiology scans are employed for diagnosis objectives in patients with pulmonary illness due to the inadequate specificity of RT-PCR. Principal chest digitized X-ray technologies are still beneficial since they are quicker, produce a reduced radiation dose, are less costly, and are widespread [4,10]. To increase the accuracy of COVID-19 diagnosing, scans or X-rays must be performed frequently in conjunction with RT-PCR data [10]. On the other hand, the considerable proportion of persons that screened positive for COVID-19 makes regular testing difficult for clinicians. As a result, authorities pushed specialists and investigators to use artificial intelligence (AI) approaches to battle the COVID-19 outbreak [1].

Utilizing digitized X-ray scans, deep learning DSS implementations have effectively predicted many health conditions, including breast cancer [14,15], skin cancer [16], and pulmonary sickness [10]. The fast proliferation of the COVID-19 pandemic, which has resulted in the life-loss of millions of people throughout the globe, necessitates the use of deep learning technology to design a DSS architecture that can enhance diagnostic accuracy. This has been the driving force behind the development of a deep learning DSS system for diagnosing COVID-19 using digitalized X-ray scans.

Further, studies have indicated that individuals with COVID-19 infection might produce pneumothorax [17]. COVID-19 instances needing hospitalization have been known to be complicated by pneumothorax. With several plausible processes behind this link, establishing the association involving pneumothorax and COVID-19 is difficult [18]. Pneumothorax has been considered an adverse prognostic indicator in COVID-19 illness [19,20]. As a result, physicians should know that a pneumothorax might be seen in the scans and physiological symptoms of COVID-19 and that this can result in a rise in fatality or severity. If a person has COVID-19 and is misdiagnosed as pneumothorax, he/she will not be in quarantine anymore, which may result in another chain of people getting infected. In cases when a person having pneumothorax is misdiagnosed as having COVID-19, he or she will still develop COVID-19 by being in touch with other patients. In both cases, another COVID-19 chain will start and will cause more COVID-19 patients, as well time and money being wasted on misdiagnosed disease.

Remote sensing can be defined as rendering information about something, without being physically present near that object. In medical fields, X-rays are generally used for remote sensing. The subject of remote sensing, which is referred as medical imaging in the medical field, is now a major topic being researched. It includes various methods such as X-ray, CAT scans, MRI, ultrasound, and endoscopy. PET and SPECT also fall under the medical remote sensing category. These various methods can produce “static” images and can be viewed in real time to track “movements” within the body. Moreover, some methods concentrate on skeletal parts, others on internal organs; others on circulation and other functions. Most methods are used to detect abnormalities such as malignant growths, bone breaks, and disease effects.

These days, researchers have created a variety of artificial intelligence (AI) algorithms for analysing medical images, such as chest X-rays, to detect infectious illnesses [21,22]. These established methods might aid medical professionals or physicians, improve the treatment test procedure, and reduce workload by automatically identifying the infection in chest X-rays. With these advanced tools and approaches, early illness diagnosis can also lower death rates. Thus, an intelligent, sensor-based health care system is proposed in the current research to classify viral illnesses, such as COVID-19 and pneumothorax, in chest X-rays, motivated by the enhanced performance outcomes of prior systems.

### Contributions

The current research is intended to be a clinical study that examines how to distinguish between COVID-19 and pneumothorax. This is motivated by the fact that a patient could develop pneumothorax after recovering from COVID, leading to misdiagnosis. The overall contribution of this paper is summarized as follows:

- This research focuses on the fact that many COVID-19 patients, after getting treated, are diagnosed with pneumothorax after a few days. Thus, an intelligent sensor-based system is required to look at the X-ray and classify it as normal or abnormal. If the X-ray contains some abnormalities, it requires further classification if the abnormality is that of COVID-19 or pneumothorax.
- We explore a variety of pretrained deep learning models and present each result compared with the proposed model. In our method, we divide the problem into two major categories, which are normal/abnormal classification and COVID-19/pneumothorax classification.
- We have proposed our final models for each classification to assist developers and researchers in broadening their perspectives on deep learning (DL) techniques and to use the models for a better purpose. Proposed DL models can be used to solve the mentioned issue accordingly.
- Finally, we point out and discuss potential reasons for our model providing better results and the future scope of this research.

The manuscript is further divided into various sections. Section 2 explores the state-of-the-art concerning COVID-19 detection from X-ray scans. Section 3 briefs the motivation behind the proposed model. Section 4 explains all the details related to the proposed model. Section 5 highlights the experiments performed and the results achieved. Finally, the research is concluded in Section 6.

## 2. Related Work

Confirmed the existence of COVID-19 in 2020, specific deep learning-based techniques are often used to identify COVID-19 on electronic X-ray and CT scans. The findings of the research performed by Kuo et al. [23] demonstrated that COVID-19 might be accurately predicted using non-image data also. To further enhance predictive performance, it is advised to add class imbalance and extraction of features while developing models for the forecasting of COVID-19. However, the current study concentrates on applying deep learning technologies to image data for the diagnosis of COVID-19 and pneumothorax. Additionally, thorough studies of the use of AI in radiological image processing for COVID-19 are offered in Fusco et al. [24].

Oh et al. reported a patch-based deep learning DSS system comprising segmentation and classification phases that would recognize COVID-19 from CXR scans in [25]. 

FCDenseNet103 has been used to split and identify the whole lung areas from the total CXR scans in segmentation. Various unrelated patches (i.e., areas of concern) are selected from such segregated lung areas and used as feed for the deep classifier network. Researchers examined CXR scans from a diverse patient population, including those who were normal and those with COVID-19-related bacterial meningitis, TB, and viral pneumonia. For the F1-score and total accuracy, respectively, predictive accuracies of 84.40% and 88.9% have been obtained.

Ozturk et al. [10] introduced the DeepCovidNet, a deep learning network that can identify COVID-19 from digitized chest X-ray scans. They used 17 CNN layers in the system to establish binary classifier (predicting COVID-19 and normal) as well as multi-class classifier (predicting COVID-19, normal, and pneumonia) predictions. For the binary and multi-class diagnoses, they attained total prediction accuracy of 98.08% and 87.02%, respectively. 

Fan et al. [26] suggested Inf-Net, a deep learning network that could detect or split dubious areas on chest CT scans that are symptomatic of COVID-19. They employed a concurrent partial decoder to build the worldwide depiction of the significant patterns. The divided borders have been enhanced using implicit reverse and edge attention. With Dice and the increased aligning score, they attained segmentation accuracies of 73.90% and 89.40%, respectively.

Wang et al. [27] developed COVID-Net using a deep learning framework to discriminate COVID-19 individuals from pneumonia patients and normal people using X-ray scans. Adopting the same set of X-ray scans, their model’s prediction results have been compared to that of VGG-19 and ResNet-50. COVID-Net beat VGG-16 and ResNet-50, according to the investigators, with positive predictive values (PPVs) of 90.50, 91.30, and 98.90% for normal, pneumonia, and COVID-19, respectively.

Based on 50 computerized chest X-ray scans, Hamdan et al. [28] proposed a deep learning COVIDXNet framework that may be utilized to identify COVID-19 patients and normal persons. Researchers evaluated the classifier performance of seven well-known convolutional models as feature extractors. VGG-19 and DensNet201 achieved the greatest analytical result of 90% compared to other classification models. 

The capacity of five well-known deep learning architectures to identify COVID-19 on electronic X-ray scans has been examined by Apostolopoulos et al. [29]. They evaluated three major categories: healthy, pneumonia, and COVID-19, with the VGG-19 classification achieving the highest accuracy rate of 93.48%. They also evaluated all the underlying models as the binary classifier (for COVID-19 vs. normal) and concluded that VGG-19 had the greatest accuracy of 98.75%. Ahuja et al. [30] presented a three-phase deep learning prediction system for binary classification to identify COVID-19 from CT scans. They employed ResNet18, ResNet50, ResNet101, and SqueezeNet as backend deep learning algorithms for data augmentation, transfer learning, and abnormality localization. The pre-trained ResNet18 using the transfer learning technique made the highest analytical findings of 99.82% (in training), 97.32% (in validation), and 99.40% (in test). 

Based on entire X-ray scans, Khan et al. [31] suggested a deep learning CNN model (i.e., CoroNet) that can be employed to assess COVID-19 as a multi-class classification issue. They could distinguish COVID-19 from bacterial pneumonia, viral pneumonia, and normal pictures with a recognition rate of 89.6%.

Using chest X-ray scans, Narin et al. [32] contrasted the classifier accuracy of three distinct deep learning CNN models (i.e., ResNet-50, InceptionV3, and InceptionResNetV2). They tested the capacity of all those proposed theories to distinguish participants with COVID-19 from those who did not have COVID-19, and ResNet-50 had the greatest classifying accuracy of 98%.

Ardakani et al. [33] compared eleven well-known DL models for detecting COVID-19 in daily medical environments on CT scans. They used a binary classifier test to distinguish between COVID-19 and non-COVID-19. ResNet-101 and Xception DL models produced the highest detection performance, with the highest accuracy of 99.40%. However, Ardakani et al. have not tested the underlying model on a diversified dataset. The current study proposes a new model that showcased better accuracy on a diversified dataset, including not only COVID-19 patients but also pneumothorax patients.

Pereira et al. [34] proposed a texture descriptor-based classifying strategy using a CNN model. They employed a resampling approach to harmonize the training set for a multi-class classifier. The prototype received a 65% F1-score. Furthermore, [33,35] give detailed survey research on deep learning approaches relevant to COVID-19. COVID-19 has been diagnosed using deep learning approaches on complete X-ray scans. This is due to a paucity of X-ray scans with identified areas of probable lesions. However, using the whole X-ray scan to make an accurate COVID-19 classification is not practicable [36].

COVID-19 has been associated with pneumothorax in a limited population; however, the relevance and prevalence of this connection are unknown. According to retrospective investigations of COVID-19 cases, pneumothorax occurs in 1% of those needing hospitalisation, 2% of those requiring ICU hospitalisation, and 1% of those passing away from the illness [37,38]. It is more complicated to comprehend the link between these diseases. Although cavitation was assumed to suggest pulmonary infarction in one case, radiology typically revealed normal COVID-19 alterations. This association might be explained in a variety of ways [39]. As a result, detecting worrisome areas related to pneumothorax disorders is crucial for obtaining a more precise test since it may be employed to extract more indicative deep aspects of the disorders.

Current research authors seek whether the patient is healed from COVID-29, has COVID-19, or has acquired pneumothorax. Numerous research works have been published in the domain of diagnosing normal and abnormal X-rays. However, to the best of our knowledge, no effort has been performed to classify COVID-19 from pneumothorax from a collection of aberrant pictures. Thus, the authors used deep learning techniques to complete the stated categorization assignment.

## 3. Motivation

Before discussing the suggested approach, we initially discuss the transfer learning gridlock for COVID-19 diagnosis based on X-ray scans, which has prevented deep learning investigators from obtaining the requisite accuracy. Let us refer to a deep neural structure as D(n~N; £), where n is a sample of the image collection N and £ is the collection of model parameters, often known as weights. The model’s training goal is to encode the range N, which is accomplished by maximizing across a huge number of observations in N. When the representative sample is limited, D has trouble accurately modelling N.

The following phase is to use transfer learning to compute the mapping Ω: Đ(y~Y; ¥)→D(n~Ɲ; £), where Đ(.) represents the pre-trained model learnt from a substantial amount of Y observations while Ɲ is a limited portion of N samples seen. Assuming a constant Ɲ, the mapping’s effectiveness is mostly determined by the distributional displacement ||Y-N||. The lower the displacement, the more indicative Đ(.) of the sample set N is, which is desirable for superior Đ(.) classification in the range of N. However, the differential displacement between ImageNet [40] coloured organic photos and X-ray scans are too high, compromising the mapping. Increasing the size of Ɲ might certainly assist since a bigger distributional displacement implies a bigger ||¥-£||, which can be explained through a more thorough representation of N in Ɲ. The authors of [41] show, however, that raising Ɲ artificially does not benefit with this job. We may conclude from our detailed examination of the topic that the data pre-processing strategies utilised in [41] cannot render Ɲ better representative of the population N. Given the impossibility of increasing Ɲ, we will concentrate on enhancing the mapping operation directly.

Assuming Ω: Đ(y~Y; ¥)→Ď(z~Ȥ; ƺ)→D(n~Ɲ; £), such that ||ƺ-£|| << ||¥-£|| and we can also estimate a fair estimation of Ď(.) by transferring Đ(.) to it, since we can organize for a higher number of observations of Ȥ. As a result, we use an interim model with a lower displacement than the output model to decrease the distribution difference between the pre-trained and the output models while also allowing for improved transfer of the pre-trained model, given the abundance of additional training data. The detail of the proposed model is provided in the next section. 

## 4. Proposed Model

**Figure 1:**
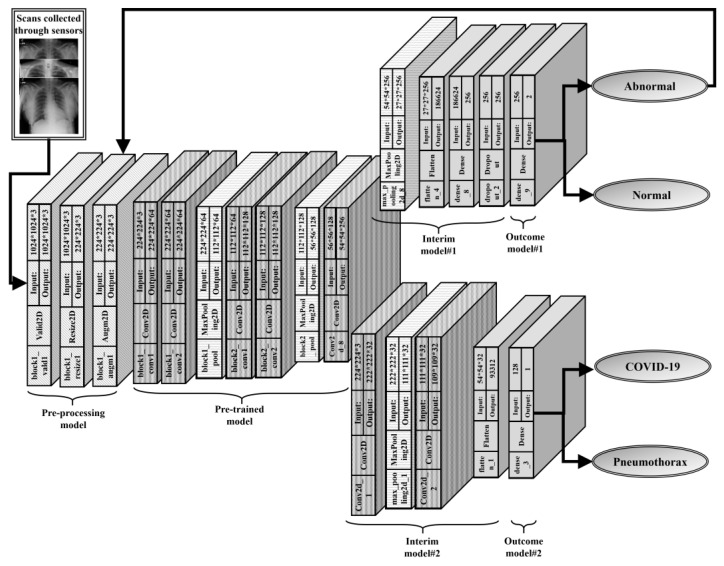
Proposed Model.

Authors attempt to determine if a patient who still suffers from COVID-19 has recovered or has acquired a pneumothorax. We divided the assignment into two parts to make it easier. To begin, the authors developed a model that can distinguish between normal and diseased X-rays (Model #1). Then, if the scans are aberrant, we have trained a second model for pneumothorax and COVID-19 classification (Model #2). The first model may be used by a developer to determine whether a patient has recovered. If the patient’s X-ray is abnormal, the scan can be sent to the second model, determining if the problem is COVID-19 or pneumothorax. We demonstrate the proposed architecture in Figure 1 and detail it further using the schematics given.

Conv2d layer is used to obtain the feature maps of images. These maps are the 2d matrix of values calculated according to the following formula: the input image is denoted by a and our kernel by b. The number of rows in the kernel is denoted as Rrow and the number of columns is denoted as Rcolumn. The indexes of rows and columns of the feature map matrix are marked with i and j respectively. The equation of the map is given by:

Map[i][j] = a[i][j] × b[i][j](1)

which gives,

(2) $$\mathrm{map}[i][j]=\sum_{x=0}^{Rrow}\sum_{y=0}^{Rcoloumn}b\;\left[x\right]\left[y\right]\;a\left[i-x\right]\left[j-y\right]\;$$

Pooling layers are then used to decrease the size of feature maps as whole maps would be time and space-consuming if used. We have used max pool as a function of the pooling layer which stores the max value of each mXn sub-matrix across the feature map.

(3) $$\mathrm{pool}[i][j]==\max\left[\begin{array}{c} \;\;\;\begin{array}{cc} \mathrm{map}\left[i-0\right]\left[j-0\right]\ldots \ldots \ldots \ldots & \mathrm{map}\left[i-0\right]\left[j-n\right]\\ \mathrm{map}\left[i-1\right]\left[j-0\right]\ldots \ldots \ldots \ldots & \mathrm{map}\left[i-1\right]\left[j-n\right] \end{array}\\ .\\ .\\ .\\ .\\ \mathrm{map}\left[i-m\right]\left[j-0\right]\ldots \ldots \ldots \ldots \;\;\;map\left[i-m\right]\left[j-n\right] \end{array}\right]$$

Dimension of feature map:

fr × fc(4)

Output after pooling:

(fr − m + 1) × (fr − n + 1)(5)

The flattened layer flattens the 2D array into a one-dimensional array. The output of the flattening matrix “arr” is given by:

arr[c] = pool [floor (c/n)] [c%n](6)

### 4.1. Pre-Processing Model

The model first looks for any abnormalities in the input chest X-ray scan. The training dataset is first down-sampled to avoid data disparity and then processed by the pre-processing model. The input X-ray images are pre-processed for classification using the pre-processing model. All X-ray scan data have been treated using conventional TCIA curation methods at the first layer of the pre-processing model. TCIA de-identifies data saved as per the Digital Imaging and Communications in Medicine (DICOM) protocol using a standards-based technique. The DICOM scans are then processed by converting them to PNG format. The photos are then downsized to 224 × 224 × 3 on the following layer. Data augmentation is used in the final layer of the pre-processing model to boost the effectiveness of deep learning techniques for limited datasets and to generate a balanced dataset. The pictures are created using three image augmentation techniques (sheer, magnification, and horizontal flip).

### 4.2. Pre-Trained Model

The real scans are utilized as the input space for the fundamental transfer learning job, which is a typical method. This model served as the foundation for the rest of the models, which were all given a new uneducated brain. This model is a CNN structure featuring two convolution filtering layers plus an additional pooling layer performed in triplicate. Then, three convolution filtering layers and one pooling layer were used twice. Finally, the design’s brain is made up of three completely linked layers, with the SoftMax outcome. The models are pre-trained using 1 million ImageNet [40] tagged pictures, with 224 × 224 × 3 colour pictures, mapped to 1000 target classes. As our pre-trained model, we have taken the top seven layers of this architecture.

### 4.3. Interim Model #1

We initially add small structural alterations to the pre-trained model while retaining its fundamental weights because our target dataset of X-ray scans contains ’large 3-channeled pictures’. We specifically changed the last levels of the original model with our own customized layers. Our technique is to employ three filters to generate a 3-channel feature map while keeping the hyper-parameters of kernel size and duration comparable to the first convolutional layer of the initial formulation. When the initial design is employed, we use the authentic activation function. To obtain the interim model, we intend to train and develop the layers as well as fine-tune the original version for an “intermediary” zone. We use chest radiology scans as our intermediary zone because they allow large-scale, Chest-Xray14 [42] categorization of normal and pathological conditions. Because they are huge medical pictures, they are more like X-ray scans, so the final layer of the interim model is changed to forecast those categories. The output of this model is the same as previous dense layer input with random nodes converted to zero value. We will consider this output as O_11_.

### 4.4. Outcome Model #1

We employ 224 × 224 × 3 inputs received from pre-processing the X-ray pictures to transfer the intermediate model to the outcome sphere of X-ray scans, a final dense layer with a “softmax” activation function. Aside from the benefit of transferring a model of diagnostic pictures to the X-ray sphere, we can also employ a greater input size. This is advantageous since bigger visuals carry richer data, resulting in more distinct patterns. With the 3-channeled pictures from the outcome region, we have a pre-trained model after fine-tuning the interim model for 100 epochs. This outcome model 1 gives output as a normal or abnormal X-ray. Consider the output of this model as O_12_.

(7) $$O_{12}(O_{11}{)}_{i}=e^{\mathrm{zi}}\;/\sum_{j=1}^{2}e^{\mathrm{zi}},$$

where, z_i_ = input vector, e^z^ = exponential function

O_12_(O_11_)_i_ = e^zi^/e^z1^ + e^z2^ (8)

Thus, Output1 = index of (max (O_12_)), i.e., class 1 or 2.

### 4.5. Interim Model #2

The interim model #2 receives pre-processed scans of input pictures that have been classified as abnormal by outcome model #1. The goal of this model is to differentiate between COVID-19 and pneumothorax. The resulting sphere of X-ray scans has been processed using VGG16 layers in a pre-trained model and additional layers with a predefined configuration as interim model 2. When it comes to distinguishing COVID-19 from pneumothorax, transfer learning performs better in comparison to classifying normal/abnormal. This is due to large number of abnormalities in abnormal sections. We have also used a huge picture size. This is advantageous since bigger pictures contain richer data, resulting in more distinct patterns. The pooling layer, also known as the subsampling layer, is a crucial component of CNN. The pooling layer functions autonomously on each feature map derived by the convolution layer. It reduces the geographical size of the feature map and delivers the essential components to reduce overfitting and the density of feature sets. In the CNN model, pooling might be the maximum, mean, or aggregate. Since others will not be able to spot the acute characteristics as readily as max pooling, it was employed in this investigation. To balance the input layer and accelerate the learning operation among hidden layers, the approach modifies the scale and activation. Consider the output of this model as O_21_.

(9) $$O_{21}(z{)}_{i}=e^{\mathrm{zi}}\;/\sum_{j=1}^{128}e^{\mathrm{zi}},$$

### 4.6. Outcome Model #2

The loss function employed is categorical cross-entropy, and the optimizer is “Adam.” For the final result, we used the “softmax” activation function. This last layer is the secondary target component since it identifies the type of abnormality (i.e., COVID-19 or pneumothorax in our case). After flattening, the vector data (from interim model #2) are fed into the CNN’s subsequent layers, known as fully connected or dense layers. Every neuron in the preceding layers is directly linked to each of the neurons in the following layer in a completely connected structure. Dense layers’ main role is to accept the flattened outcome of the convolution and pooling layers as input and categories the picture to a specified class label.

O_2__2_(O_2__1_) = 1/1 + e−^O_21_^, (10)

Output2 = 1, if O_22_ > 0.5, else Output2 = 0

## 5. Experimental and Results Section

### 5.1. Dataset

**Figure 2:**
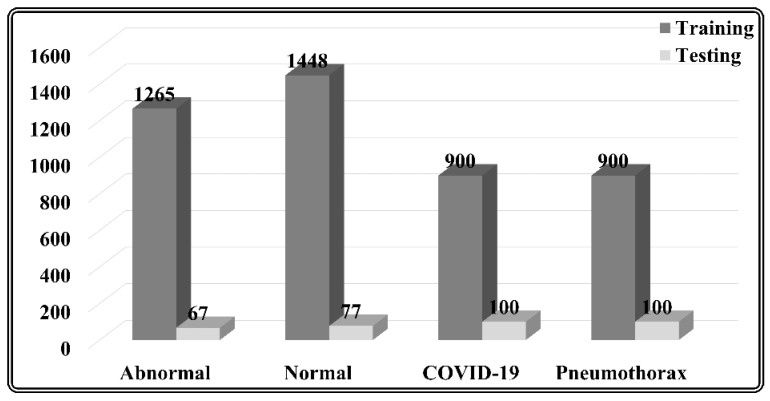
Dataset Distribution.

The experimental analysis of the proposed model has been conducted using a blend of three datasets, i.e., NIH chest X-ray dataset [42], SIIM dataset [43], and CDI COVID-19 dataset [44]. The dataset crafted using the “NIH chest X-ray dataset” distinguishes normal and abnormal X-rays. Anyone with no abnormalities has been classified as normal, whereas scans with exhibiting abnormalities have been classified as abnormal. A total of 1265 aberrant scans were chosen at random for training, and 67 aberrant scans were selected at random for testing. The authors acquired 1448 scans for training and 77 images for testing for normal. Further, the authors used two distinct datasets to diagnose COVID-19 and pneumothorax. For training, the authors selected 900 random scans from the “SIIM dataset” and “CDI COVID-19 dataset,” which includes scans for pneumothorax and COVID-19, respectively, and 100 scans for testing. Figure 2 depicts the distribution of data belonging to distinct classes.

### 5.2. Performance Evaluation Metrics

To investigate the categorization of COVID-19 and pneumothorax sufferers, the authors use the datasets supplied for X-ray scans to evaluate the performance of the proposed models. The authors highlight four outcomes that are characteristic of CNNs for every framework:

- Accuracy Curve.
- Loss Curve.
- Confusion Matrix.
- Area Under Curve (AUC).

The model’s training and validation accuracy curves illustrate how effectively the model is learning/summarising. Overfitting is measured by the difference in training and validation accuracy. The loss curve depicts the learning phase and the orientation of the model. The learning scope with training is depicted by a large gap between the training and validation arcs. A confusion matrix is a tool that describes a classifier’s effectiveness in a collection of the testing dataset for which the actual values are previously known. Every confusion matrix has four fundamental words linked with it [45]. (i) True Positives (TP): these are situations when it is forecast “yes”, and, indeed, the affected person had the condition. (ii) True Negatives (TN): it is estimated “no,” and they are not infected. (iii) False Positives (FP): It is anticipated a “yes” for the condition, but the sufferers do not have it. This is sometimes referred to as a Type I error. (iv) False Negatives (FN): When the model suggests “no,” people nonetheless have the condition. Type II errors are what they are labelled. It is often used to depict essential prediction statistics, making it simpler to analyse and obtain useful experimental patterns. AUC is a composite performance indicator that considers all possible categorization levels. The likelihood that the model rates an arbitrary favourable instance higher than a random counter example is one approach to analyse AUC. The AUC represents the likelihood of an arbitrary favourable instance being placed to the right of a randomized counter-example. The AUC value varies from 0 to 1. The AUC of a model for whom forecasts are 100% incorrect is 0.0, whereas the AUC of a model where forecasts are 100% accurate is 1.0.

### 5.3. Environmental Setup

The experimentation was split into two possibilities. The authors employed a modified VGG16 in the first case (for interim model #1) and another modified VGG16 in the latter (for interim model #2). Over the first setting, scans of normal subjects were combined with scans of patients with anomalies to create a classification engine. In the later trial, the authors developed a model to distinguish COVID-19 from pneumothorax in patients with aberrant X-ray scans. Python language has been used to simulate these two situations. The authors utilized an open-source deep learning approach to build the systems, methods, modules, and materials of TensorFlow 2.0 (plus Keras, Google, Mountain View, CA, USA). The analyses have been carried out with the help of Python. All tests have been performed on Google Collaboratory with a Tesla K80 GPU graphics card(NVIDIA, Santa Clara, CA, USA), an Intel (Santa Clara, CA, USA) i7-core @3.6 GHz CPU, and 16 GB RAM on a 64-bit Windows 11 operating system (Microsoft, Redmond, Washington, DC, USA). To evaluate the performance of the proposed model, the authors performed a series of tests on the identical datasets, feeding them into the two most adopted pre-trained CNN models: InceptionV3 and Resnet50, including a different configuration of VGG16 of fixed layers and learnable convolution modules. Additionally, the authors selected each model’s excellent score independently and then assessed every model on a testing dataset.

### 5.4. Results

#### 5.4.1. Model Forecasting Normal/Abnormal

The effectiveness of the various pre-trained CNN models (as recommended in [32]) and the proposed model is summarized in Table 1. It is observed that almost all of the tested pre-trained models did an excellent job of categorizing normal and abnormal photos. While the findings of the other CNNs appeared only somewhat different, the proposed model showcased the best accuracy of 89.58%. In terms of accuracy and AUC, the proposed model beat alternative models.

**Figure 3:**
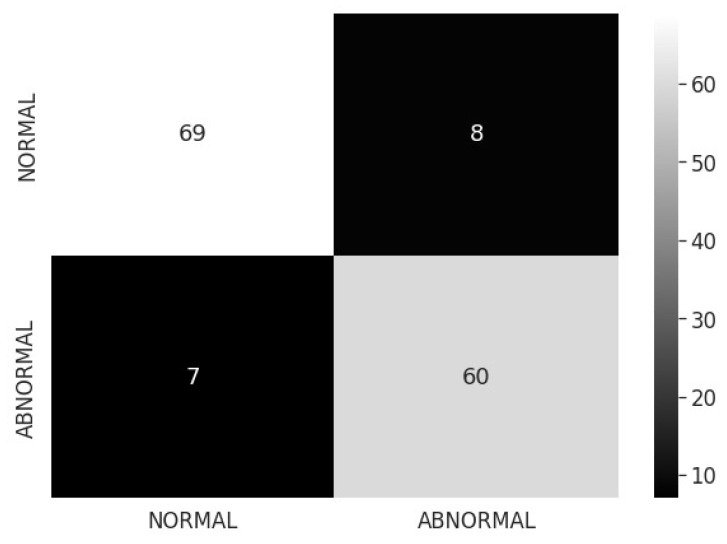
Confusion matrix for proposed model normal/abnormal classification.

**Figure 4:**
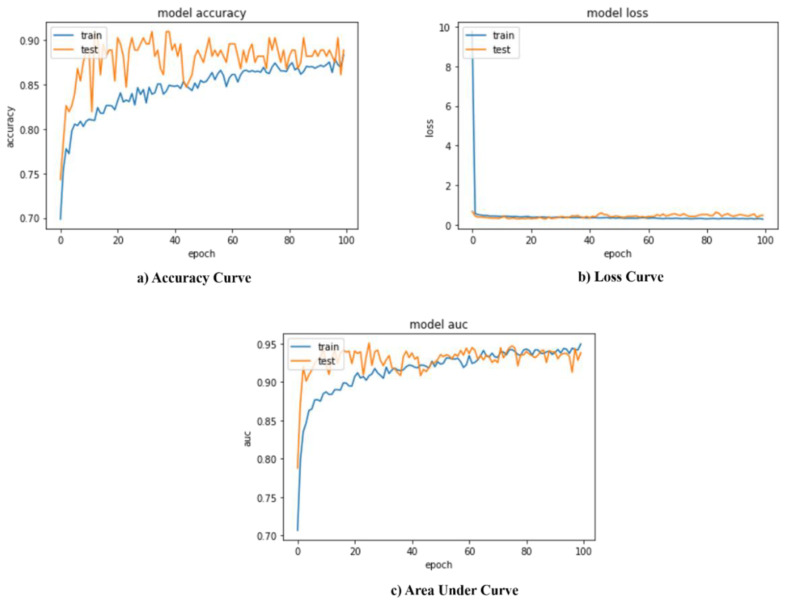
Performance evaluation metrics of proposed model normal/abnormal classification.

The confusion matrix for two-class classification with normal/abnormal is shown in Figure 3. The suggested model’s training and validation accuracy, loss, and AUC are shown in Figure 4. The model converges successfully, as can be noticed because the gap in both the train and validation arcs is minimal.

#### 5.4.2. Model Forecasting COVID-19/Pneumothorax

Table 2 summarises the effectiveness of each of the several pre-trained CNN models. Almost all the examined pre-trained models performed admirably in identifying COVID-19 and pneumothorax scans. While the results of the other CNNs appeared to differ slightly, the suggested model had the highest accuracy of 99.5%. The presented model outperformed competing models in terms of accuracy.

**Figure 5:**
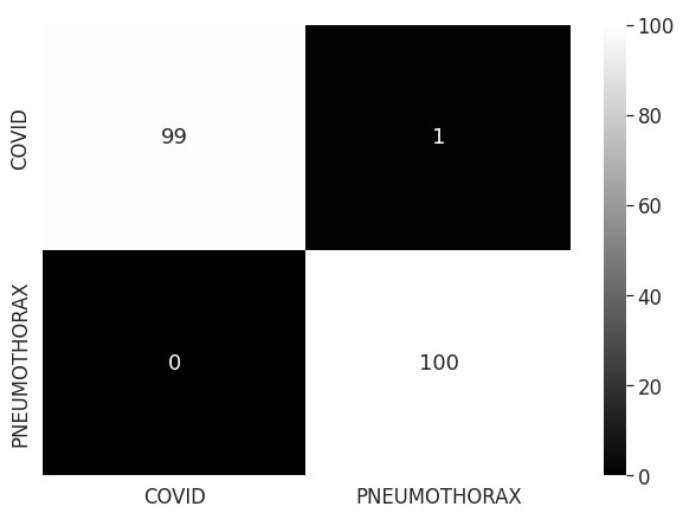
Confusion matrix for proposed model COVID-19/pneumothorax classification.

**Figure 6:**
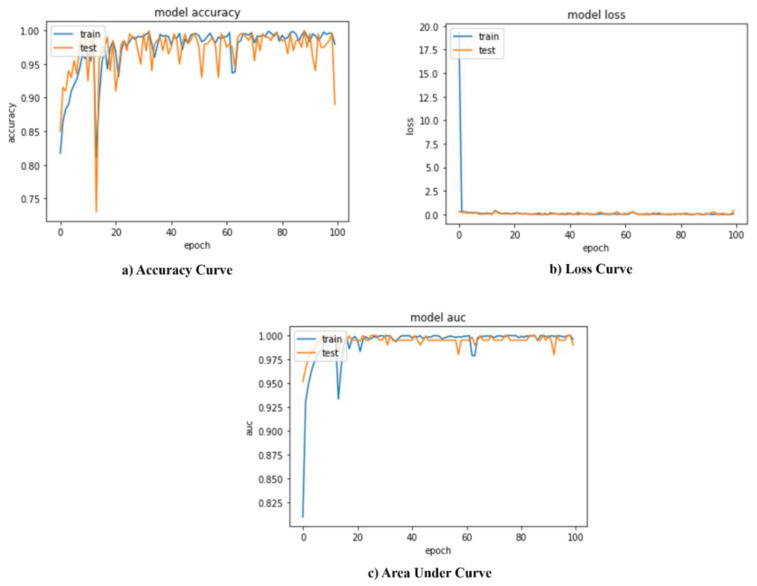
Performance evaluation metrics of proposed model COVID-19/pneumothorax classification.

Figure 5 depicts the confusion matrix for COVID-19/pneumothorax two-class categorization. Figure 6 shows the recommended model’s training and validation accuracy, loss, and AUC. The model converges well, as evidenced by the small gap between the train and validation arcs.

#### 5.4.3. Comparison with Existing Classifiers

**Figure 7:**
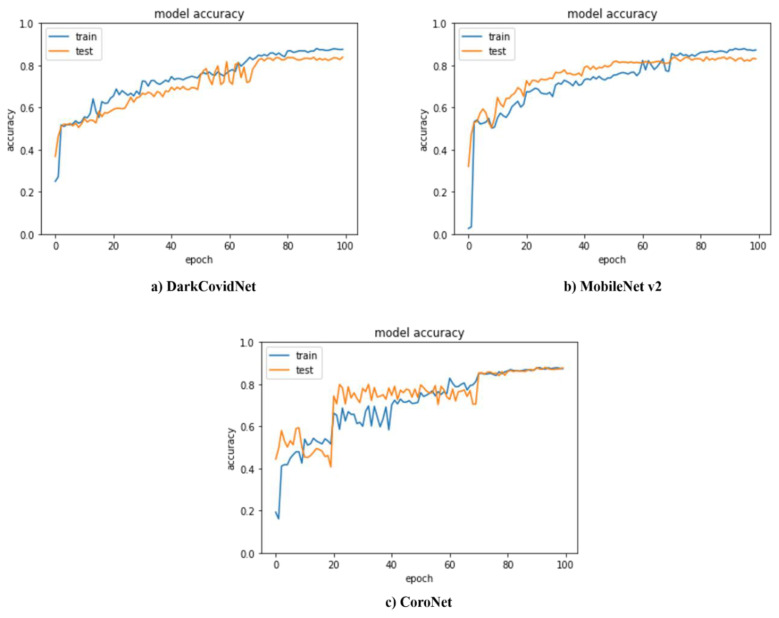
Accuracy of various state-of-the-art classifiers.

The primary motivation behind the current research is to pioneer the deep learning application for pneumothorax patients. The existing literature [10,31,46] demonstrates three class classifiers for COVID-19, Normal, and Pneumonia classification. Focusing on the aim of the current research, we adapted the three class classifiers suggested in references [10,31,46] with the diversified dataset constituting scans for normal, COVID-19, and pneumothorax patients. The accuracy achieved from the underlying three classifiers is presented in Table 3 and Figure 7. The proposed model exhibits the highest accuracy, i.e., 99.5% (Stage 2: COVID-19/pneumothorax) of 89.58% (Stage 1: Normal/Abnormal), which equals 89.13%.

Further, it has been observed that the proposed model produces the results at a speed of 95 frames per second (FPS) for both models, which is near to real-time (as depicted in Table 3.). The proposed model’s speed is recorded slightly lower than other models under consideration due to adopting two-stage classifiers instead of a single three-class classifier. However, the slight speed difference in speed can be compromised to attain better accuracy.

## 6. Discussion

The suggested approach automatically identifies COVID-19 and pneumothorax in X-ray scans without needing customized feature extraction methods. Experts at treatment centres can use the proposed methodology to obtain a consultation. It can considerably reduce clinician effort while also assisting them in making correct diagnoses in their everyday activities. Since the suggested method saves time (the diagnosis procedure is quick), professionals may devote their attention to more urgent situations.

Initially, the model is tested for classifying normal and abnormal scans on a batch of 144 scans with equal amounts of each classification. The proposed model has been able to distinguish normal and abnormal chest X-rays with an accuracy of 89.58%. A test set of 200 scans has been used to test the model’s capability to identify COVID-19 and pneumothorax, which uses abnormal scans as input. It correctly classified COVID-19 and pneumothorax with an accuracy of 99.5%. As a result, the accuracy of accurately identifying normal pictures is 89.58%, while correctly predicting COVID-19 and pneumothorax is 99.5% of 89.58% or 89.13%.

This work showed that deep learning combined with X-ray scans could identify important biological indicators linked to COVID-19 and pneumothorax illness. However, there are certain flaws in this research. Since a unified dataset for this job is still unavailable, several datasets have been blended to create a single dataset to discriminate between COVID-19 and pneumothorax. However, we employed DICOM pictures, which is a standard format. The scans for the two classes may have been conditioned differently because the goals for both original datasets are separate. A more in-depth investigation may be carried out if a dataset is created for this specific work, which would considerably enhance the task and make it more dependable. The presented work can be extended to other fields, such as the classification of land cover types [47,48,49,50].

## 7. Conclusions

Various pre-trained deep learning models were used in this research to select the optimum deep learning strategy for differentiating normal from abnormal X-rays and COVID-19 from pneumothorax. Using the aforementioned dataset, several tests were carried out to determine which layer is capable of extracting the greatest characteristics and achieving the best results. Deep networks outperformed other systems in all measures when it came to identifying normal from abnormal X-rays, as well as COVID-19 and pneumothorax, especially the proposed model, which was constructed employing transfer learning by VGG16 and outperformed other models (Resnet50 and InceptionV3) in all parameters. The classification accuracy, loss, and AUC of normal and abnormal X-rays are 89.58%, 0.25, and 95%, respectively. The classification accuracy, loss, and AUC of COVID-19 and pneumothorax X-rays are 99.5%, 0.01, and 99.5%, respectively. As a result, the accuracy of accurately identifying normal scans is 89.58%, whereas for forecasting COVID-19 and pneumothorax is 99.5% of 89.58%, i.e., 89.13%.

## Figures and Tables

**Table 1:** Performance of various models for forecasting Normal/Abnormal.

Model	Accuracy (with 95% CI)	AUC (with 95% CI)	Loss
InceptionV3 [32]	86.2 ± 0.052	0.95 ± 0.0955	0.34
Resnet50 [32]	88.4 ± 0.05	0.92 ± 0.1188	0.28
Proposed Model	89.58 ± 0.049	0.95 ± 0.0955	0.25

**Table 2:** Performance of various models for forecasting COVID-19/pneumothorax.

Model	Accuracy (with 95% CI)	AUC (with 95% CI)	Loss
InceptionV3 [32]	99.1 ± 0.0975	0.995 ± 0.0112	0.02
Resnet50 [32]	98.4 ± 0.0980	0.994 ± 0.0126	0.01
Proposed Model	99.5 ± 0.0970	0.995 ± 0.0112	0.01

**Table 3:** Accuracy and speed comparison with state-of-the-art classifiers.

Model	Accuracy	FPS
DarkCOVID-Net [10]	84.2%	99
MobileNet v2 [46]	86.1%	97
CoroNet [31]	88.7%	94
Proposed Model	89.13%	95

## Data Availability

The data presented in this study are openly available in [42,43,44].
